# Supplementary material for: Asymmetric Synthesis of Quaternary Hydantoins via a Palladium-Catalyzed Aza-Heck Cyclization
Source: J Am Chem Soc. 2025 Nov 14;147(49):44692–8. doi: 10.1021/jacs.5c16022 (PMC12703750; doi:10.1021/jacs.5c16022)
Supplement: Supplementary file 2 [file ja5c16022_si_002.zip › All NMR FID Files/S8 After Heating/S8-A_ProtonNMR/1/pdata/1/email_EM06-036-isomer#1-dry-1H_1_1.pdf]

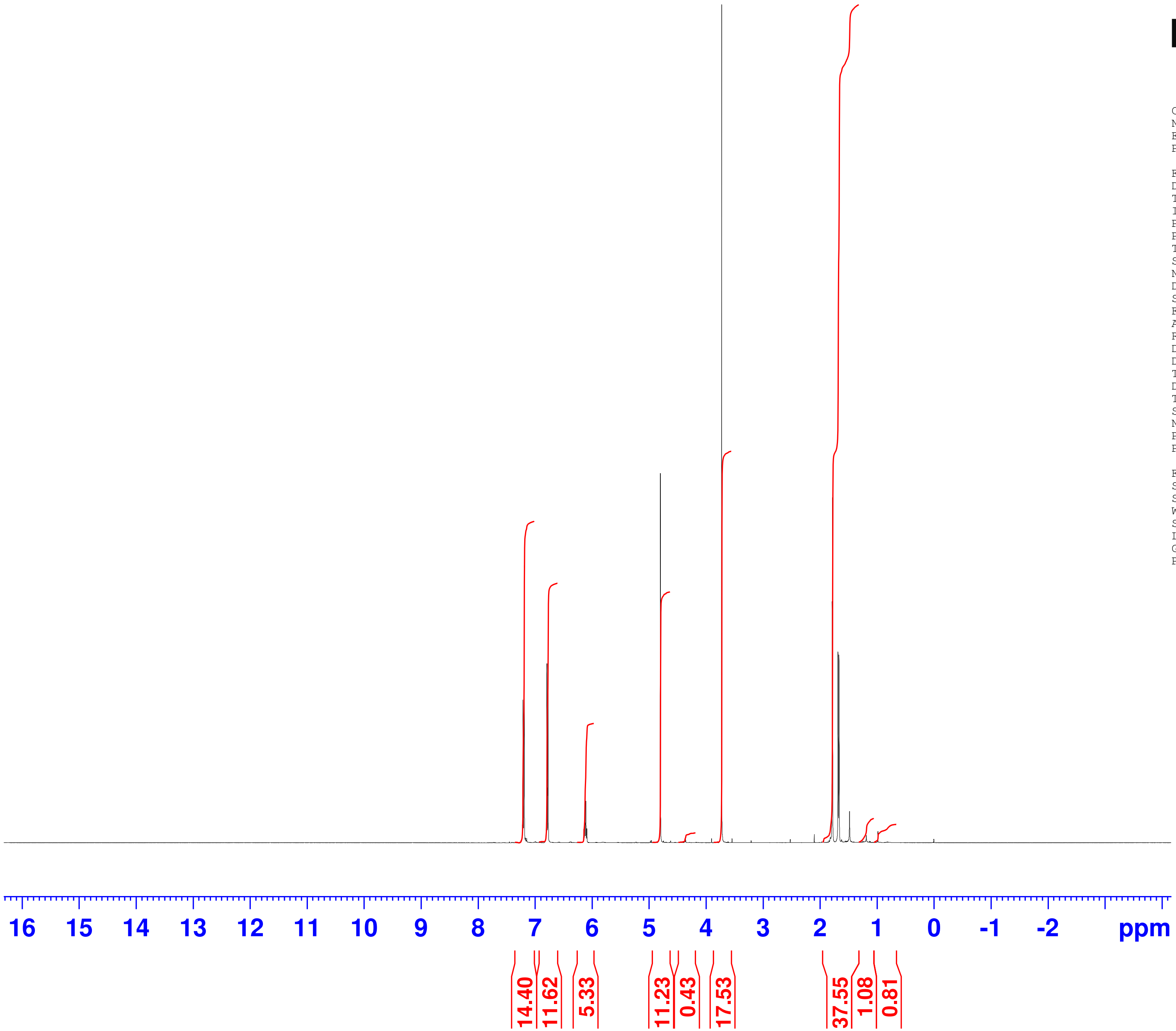

Current Data Parameters

NAME	EM06-036-isomer#1-dry-1H
EXPNO	1
PROCNO	1

F2 - Acquisition Parameters

Date_	20250211
Time	8.26 h
INSTRUM	Avance Neo 400 U of DE Brown Lab
PROBHD	Z104450_0412 (
PULPROG	zg
TD	65536
SOLVENT	CDCl3
NS	32
DS	2
SWH	8196.721 Hz
FIDRES	0.250144 Hz
AQ	3.9976959 sec
RG	101
DW	61.000 usec
DE	13.33 usec
TE	298.0 K
D1	10.00000000 sec
TD0	1
SFO1	400.1324708 MHz
NUC1	1H
P1	15.00 usec
PLW1	8.31639957 W

F2 - Processing parameters

SI	65536
SF	400.1300380 MHz
WDW	EM
SSB	0
LB	0.30 Hz
GB	0
PC	1.00
